# Enhanced Epoxy Composites Reinforced by 3D-Aligned Aluminum Borate Nanowhiskers

**DOI:** 10.3390/ma17194727

**Published:** 2024-09-26

**Authors:** Hyunseung Song, Kiho Song, Haejin Hwang, Changui Ahn

**Affiliations:** 1 Engineering Ceramic Center, Korea Institute of Ceramic Engineering & Technology (KICET), Icheon 17303, Republic of Korea; 2 Department of Materials Science and Engineering, Inha University, Incheon 22212, Republic of Korea

**Keywords:** aluminum borate nanowhisker, nanocomposites, three-dimensionally aligned, mechanical properties, protective material, portable electronic devices

## Abstract

Recently, the durability of high-performance and multifunctional portable electronic devices such as smartphones and tablets, has become an important issue. Electronic device housing, which protects internal components from external stimuli, such as vibration, shock, and electrical hazards, is essential for resolving durability issues. Therefore, the materials used for electronic device housing must possess good mechanical and electrical insulating properties. Herein, we propose a novel high-strength polymer nanocomposite based on 3D-aligned aluminum borate nanowhisker (ABOw) structures. ABOw was synthesized using a facile hydrothermal method, and 3D-aligned ABOw structures were fabricated using a freeze-casting process. The 3D-aligned ABOw/epoxy composites consist of repetitively layered structures, and the microstructures of these composites are controlled by the filler content. The developed 3D-aligned ABOw/epoxy composite had a compressive strength 56.72% higher than that of pure epoxy, indicating that it can provide high durability when applied as a protective material for portable electronic devices.

## 1. Introduction

High-performance and multifunctional portable electronic devices, such as smartphones, tablets, and wearable devices, have become essential elements in human daily life due to their convenience and efficiency. For this reason, the compound annual growth rate (CAGR) of the portable electronic market over the past 10 years has been 15.51%, and it is expected to grow to 847.88 billion USD by 2032. The importance of the lifespan and durability of electronic devices has increased owing to their high selling prices, resulting from the high degree of integration of their internal components; therefore, interest in the development of high-performance protective materials that protect the products from external forces such as shock and vibration is increasing [1,2,3,4]. Protective materials for portable electronic devices must have good mechanical properties to protect the product from external impacts and good insulating properties to ensure electrical safety [5]. In addition, high signal permeability is required because current electronic devices perform many functions using wireless signals [6,7]. The most commonly used protective materials in portable electronic devices are aluminum (Al) and glass. Al has relatively good mechanical properties and glass materials exhibit high electrical insulation and signal transparency; therefore, they are widely used as protective materials in portable electronic devices [8,9,10]. Polymeric materials have a low density of 1–1.2 g/cm^3^ compared to the densities of Al (2.7 g/cm^3^) and glass (2.8 g/cm^3^). In addition, they are lightweight, cost-efficient, and exhibit a high impact resistance (200–300 J/m). Moreover, because it is easy to control and select the type and content of the added filler, the thermal, electrical, and mechanical properties can be improved, making this system suitable as a protective material in portable electronic devices [11].

Ceramic-based polymer composites are promising protective materials because their properties (i.e., mechanical, thermal, and electrical) can be controlled by selecting the type and content of fillers and matrix materials [12,13]. Ceramic fillers include carbides, nitrides, and oxides. Carbide materials (Silicon carbide, Tungsten carbide, and Titanium carbide) have high mechanical strength and excellent thermal stability but have poor insulation due to their relatively high electrical conductivity [14,15,16,17]. Nitride materials (Aluminum nitride and Boron nitride) have good insulating properties but their manufacturing processes are difficult and complex, making them expensive [18,19]. Traditional oxide materials (Aluminum oxide, Titanium oxide, and Silicon dioxide) have low raw material costs, moderate mechanical properties, and good electrical insulation [20,21,22,23]. In particular, aluminum borate nanowhiskers (ABOw) have the advantages of a high aspect ratio, insulating properties, and good mechanical strength compared to conventional oxide materials [24,25,26]. These properties suggest that ABOw filler materials are the most efficient candidates for reinforcing polymer matrices. Another method for strengthening a polymer matrix is to uniformly distribute and connect the fillers within the matrix over the entire surface area to maximize the stress distribution effect within the composite. Composite materials manufactured by simply mixing fillers and matrices, a conventional method, have significant limitations arising from the deterioration of mechanical properties due to the uneven distribution and agglomeration of fillers [27,28]. Although various 3D structuring methods for ceramic filler materials, such as direct laser writing [29], proximity-field nanopatterning [30], and additive manufacturing [31], have been proposed, their application in protective materials for portable electronic devices is hindered by critical issues, including complex manufacturing processes, high manufacturing costs, and narrow manufacturing areas.

In this paper, we propose a new type of polymer composite with a 3D-aligned ABO nanowhisker structure for protecting portable electronic devices. ABO nanowhiskers were synthesized via a facile hydrothermal synthesis method and were uniformly aligned using a simple freeze-casting process to form a high-density, connected, and distributed structure without agglomeration, and then filled with epoxy to fabricate composite materials. The 3D-aligned ABO nanowhiskers within the polymer composite effectively transfer stress from the matrix to the strong filler materials and optimize stress distribution to maximize fracture resistance. When the filler content of the ABO nanowhiskers was 2.7 vol%, the composite exhibited a high compressive strength exceeding that of pure epoxy by 56.72%. We expect that this developed composite material will effectively protect portable electronic devices from external shocks and stimuli, extending their lifespan and improving durability.

## 2. Experimental

### 2.1. Fabrication of ABO Nanowhiskers

AlCl_3_·6H_2_O (aluminum chloride hexahydrate, Sigma–Aldrich, St. Louis, MO, USA) (24 g) and H_3_BO_3_ (6.183 g) were dissolved in 200 mL of distilled water at 90 °C with mechanical stirring. NH_4_·OH (ammonium hydroxide, Popular Chemical) (2.7 g) and NaOH (sodium hydroxide, Sigma–Aldrich, USA) (2 g) were dissolved in 96.8 mL of distilled water at 50 °C with mechanical stirring. The precipitating agent was then slowly added to the mixture, and the solvent was evaporated using an oil bath at 200 °C for 2 h. The resulting precipitate was heated in a tube furnace at a rate of 5 °C/min to 1000 °C under a N_2_ atmosphere. The obtained material was filtered and washed with distilled water to prepare a final powder, which was subsequently dried in a vacuum furnace at 90 °C for 5 h.

### 2.2. Fabrication of Densely Aligned ABOw Structures

ABOw at contents of 4, 5, and 6 wt% were dissolved in 80 g of distilled water, and 1.5 wt% NaOH relative to the total solvent and 10 wt% dispersant (DisperBYK-2019, BYK, Berlin, Germany) relative to solid content were used to achieve complete dispersion. Subsequently, 10 wt% binder (HS-BD) relative to the solid content was added, and the mixture was mechanically stirred at room temperature for 24 h. The solution was then cast into a Teflon mold using the freeze-casting method. Cu rods (−80 °C) were cooled in liquid nitrogen (−196 °C). The Teflon mold was tightly attached to the Cu rod, and the sample was frozen on the surface of the Cu rod. After freezing, the samples were dried in a freeze-dryer (Ilshin Biobase, Dongducheon, Republic of Korea) for 24 h. Finally, the resulting structure was sintered at a rate of 1 °C/min to 450 °C in an electric furnace and then heat-treated for 5 h.

### 2.3. Fabrication of ABOw/Epoxy Composites

The epoxy resin and hardener (EpoFix) (Bisphenol A, Struers, Co., Ballerup, Denmark) were mixed at 25 °C at a weight ratio of 10:1. The ABOw structures prepared for each content sample were placed in the mixed epoxy, and the air inside the structures was removed using a vacuum desiccator for at least 8 h to allow epoxy infiltration. Finally, the epoxy was cured at 90 °C for 3 h, after which the fabrication of the ABOw/epoxy composite was complete.

### 2.4. Characterization

The crystal structure of the synthesized ABOw was analyzed using X-ray diffraction (XRD, max 2500, Rigaku, Tokyo, Japan). The morphology, 3D structure, and composite form of ABOw were examined using field-emission transmission electron microscopy (JSM-7619F, JEOL, Tokyo, Japan). The crystal planes and shapes of ABOw were examined using a high-resolution transmission electron microscope (TEM, Tecnai, FEI, Hillsboro, OR, USA). For each sample, five rod-shaped specimens were prepared according to ASTM-D695 (12.7 × 12.7 × 25.4 mm^3^), and the compressive strength was measured at 500 N using a universal tester (UNITITH-T, R&B, Daejeon, Republic of Korea). The volume resistivity of the composites was measured at 500 V/mm using a high-resistance ammeter (2985B, Keysight, Santa Rosa, CA, USA) after placing the samples in a heatable Faraday cage. The data obtained from the experiment were processed using the origin pro program (OriginLab 2021).

## 3. Results and Discussion

**Figure 1:**
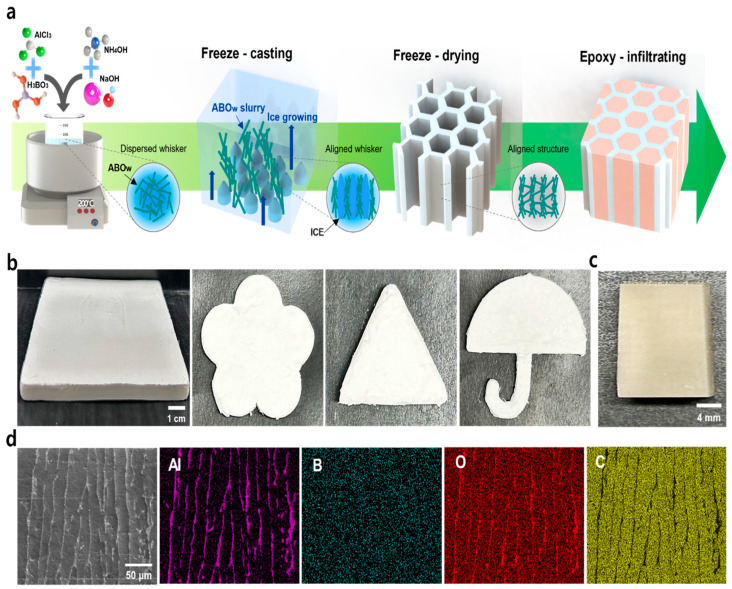
(**a**) Schematic of the fabrication of aligned ABOw structure-based epoxy composites; (**b**) optical images of the aligned ABOw structures with complex shapes; (**c**) optical image of the ABOw/epoxy composite; (**d**) EDS mapping images of the aligned ABOw/epoxy composite.

Figure 1a shows a schematic of the overall process for realizing 3D-connected structure-based epoxy composites. ABO nanowhiskers prepared via facile hydrothermal synthesis were added to distilled water, and NaOH was then added to adjust the pH for producing an ABOw slurry with high dispersion stability. Through freeze-casting, the fillers were aligned along the ice crystal path in an aqueous solution, and the ice was then sublimated to form the final aligned ABO 3D structures via freeze-drying. The aligned ABO structures were heat-treated at 450 °C to remove the added binder and strengthen the binding force between the fillers. In addition, to minimize the number of micropores inside the epoxy composites, they were manufactured by infiltrating the epoxy under vacuum. Figure 1b presents optical images of the 3D ABOw structures fabricated with a size of 10 × 10 cm^2^ and various shapes. The proposed process is highly applicable to large-area composite materials for various protective applications. Figure 1c,d show optical and cross-sectional energy-dispersive X-ray spectroscopy (EDS) mapping images of the fabricated ABOw/epoxy composite, indicating the regularly and repeatedly arranged structures of the ABOw filler materials and epoxy resin. The results confirm the effectiveness of the developed composite for protecting portable electronic devices from external shocks and stimuli by ensuring high dispersibility and connectivity.

**Figure 2:**
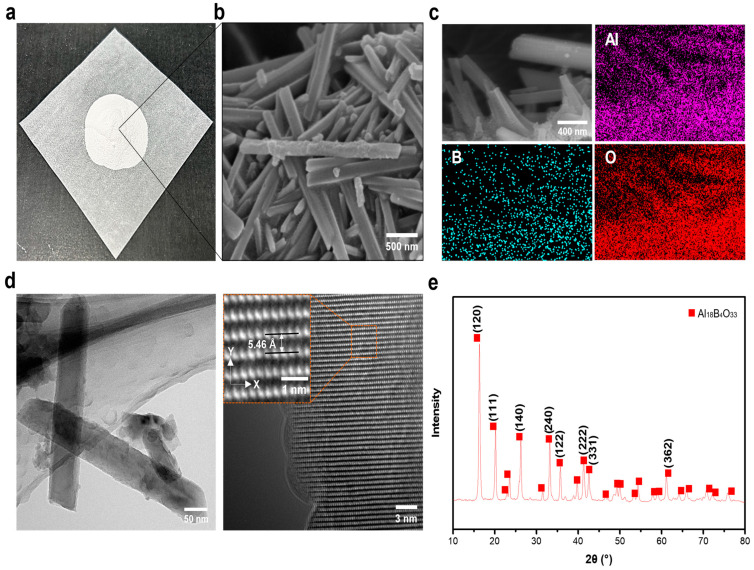
(**a**) Optical image of the fabricated ABOw; (**b**) FE-SEM image of ABOw; (**c**) EDS mapping images of ABOw; (**d**) high-resolution TEM images and the crystalline structure of ABOw; (**e**) XRD pattern of ABOw.

ABOw was produced using a facile hydrothermal synthesis method. When the precipitant (NaOH or NH_4_OH) was slowly added to a uniformly mixed aqueous solution of AlCl_3_ and H_3_BO_3_, nanowhisker-shaped precipitates were formed. These precipitates simultaneously underwent a phase change to ABOw, with the removal of unreacted H_3_BO_3_ and NaOH residues from the surface during a single heat treatment. The resulting ABOw was a uniform nanowhisker-shaped white powder with an aspect ratio of approximately 10 (Figure 2a,b). The EDS mapping results in Figure 2c confirm that Al, B, and O were uniformly distributed throughout the fabricated ABOw. As indicated by the TEM image in Figure 2d, the synthesized ABOw consisted of rod-like particles with a regularly arranged lattice. In addition, XRD analysis of ABOw indicated that it was a multicrystalline phase with a dominantly oriented (120) plane.

**Figure 3:**
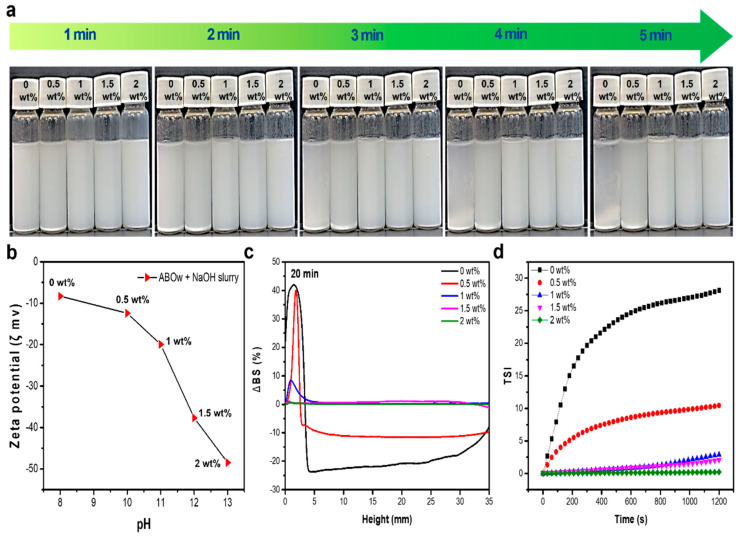
(**a**) Optical images of ABOw slurries with various amounts of added NaOH over time; (**b**) zeta potentials for various amounts of added NaOH; (**c**) Turbiscan analysis of ABOw slurries with various amounts of added NaOH (holding time: 20 min); (**d**) TSI profiles of ABOw slurries over time for various amounts of added NaOH.

The freeze-casting process used in this study was significantly affected by the particle dispersion within the slurry, consisting of ceramic particles, organic binders, and water. The dispersion stability of the ABOw slurry was low owing to the sedimentation phenomena caused by the aggregation of nanowhiskers with a large specific surface area. To address this dispersion problem, the pH of the slurry was adjusted by adding NaOH. The dispersion stability of ABOw slurries was observed over time by adding 0.5, 1, 1.5, and 2 wt% NaOH to the slurry with a filler content of 6 wt% (Figure 3a). Slurries with 0, 0.5, and 1 wt% NaOH accumulated sediment at the bottom of the solvent over time. This precipitation phenomenon causes non-uniform alignment and connection problems of the fillers during freeze-casting, which can result in poor mechanical properties of the ABOw/epoxy composites. Figure 3b shows the zeta potential with respect to pH. The absolute value of the zeta potential increased as the amount of NaOH added increased, indicating an improvement in the dispersion stability of the slurry. When strong alkaline NaOH dissolves in the solvent, dissociated OH- ions impart a negative charge to the whisker surface, increasing the surface charge of the whisker particles. Accordingly, the electrostatic repulsion between the particles increases, preventing agglomeration and maintaining stable dispersion [32,33]. The dispersion stability of the slurry was confirmed via Turbiscan analysis (Figure 3c,d and Figure S1a–e). For NaOH addition amounts of 0, 0.5, and 1 wt%, ∆BS had large absolute values at heights of <5 mm, indicating large amount of sediment accumulation at the bottom of the slurry. Additionally, for 0–0.5 wt%, ∆BS had large absolute values at heights of 5–35 mm, confirming that the particles in the slurry were severely aggregated. The absolute values of ∆BS for NaOH addition levels of 1.5 and 2 wt% were close to zero across the range of 0–35 mm, indicating high slurry dispersion stability. The slurry dispersion stability was evaluated using the Turbiscan Stability Index (TSI), calculated as

(1) $$\mathrm{TSI}=\sum_{i}\frac{\sum_{h}\left|{scan}_{i}\left(h\right)-{scan}_{i-1}\left(h\right)\right|}{H}$$

where ${scan}_{i}\;(h)$ and ${scan}_{i-1}\;(h)$ are the values of the profile for a given scan “i” and the previous one “i−1” obtained at a given height “h”. H represents the total height of the sample. In the TSI graph, a TSI value closer to 0 indicates higher dispersion stability of the slurry [34]. Therefore, it was confirmed that high dispersion stability could be obtained by adding 1.5 and 2 wt%. However, the 2 wt% slurry has a far higher viscosity, and it is difficult to uniformly form ice crystals and porous structures during freeze-casting process, because of the increased possibility of bubbles and defects due to the reduced mobility of the slurry. Therefore, when 1.5 wt% NaOH was added to the slurry, the freeze-casting process was most stable.

**Figure 4:**
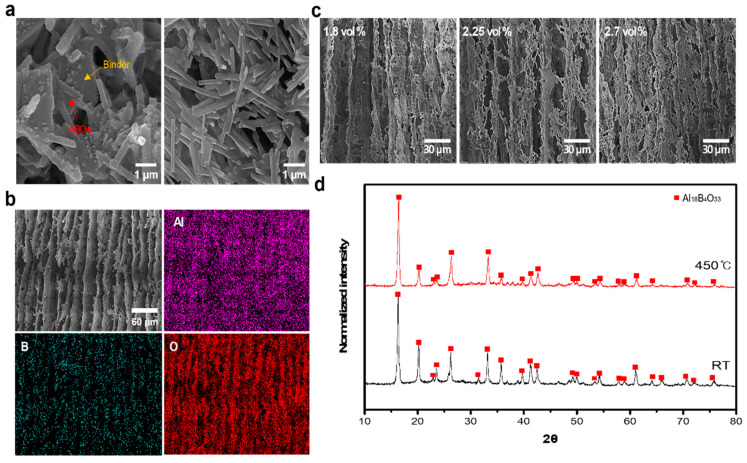
(**a**) **Left**: magnified FE-SEM image of the structure before heat treatment; **right**: magnified FE-SEM image of the structure after heat treatment; (**b**) FE-SEM image and EDS mapping of the aligned ABOw structure; (**c**) cross-sectional FE-SEM images of structures after heat treatment, with different amounts of added filler; (**d**) XRD patterns of the ABOw structure before and after heat treatment.

The 3D-aligned filler structures were fabricated using a simple freeze-casting process. Figure S2a shows that no structure formation occurs when fabricated with low filler contents. Figure S2b shows an EDS mapping image of the structure fabricated with a filler content of 2.7 vol%. The filler was uniformly distributed inside the fabricated structure, and the 3D structure was maintained even after heat treatment, as shown in Figure 4b and Figure S3a–c. Because binders and fillers have different properties, defects can occur at the interface between binder and filler particles, which may interfere with the final bonding between filler particles and cause structural discontinuity. In addition, if the binder structure is not uniform or has defects, local deformation becomes more severe. At the nanoscale, microscopic pores or defects inside the binder can cause local stress concentrations, which can be the initiation point of crack generation during the deformation process. For this reason, stress concentrations easily occur at the interface between binder and filler particles, forming microcracks. Cracks easily propagate along the interface where stress is concentrated, ultimately reducing the mechanical properties of the composite and making it prone to failure during compressive strength tests [35,36,37]. Therefore, they were effectively removed via heat treatment at 450 °C (Figure 44a,c and Figure S5a–d). After the structure was fabricated, the wall-to-wall distance in the middle region was analyzed according to the filler content (1.8, 2.25, and 2.7 vol%), and it was confirmed to be 25.31 µm when the filler content was 2.7 vol%. Accordingly, it was confirmed that as the filler content increased, the filler became more densely distributed, and a structure with high filler bonding strength was fabricated (Figure S4a). As shown in Figure 4d, XRD analysis confirmed that there was no change in the ABO phase after heat treatment.

The compressive strengths of the 3D-aligned ABOw structure-based composites were evaluated. External shocks and stimuli can damage the components or batteries inside the device; therefore, the compressive strength of the protective material is essential to prevent this and ensure stability and durability. The compressive strength can be calculated as follows:

(2) $$\sigma_{c}=\frac{F}{A},$$

**Figure 5:**
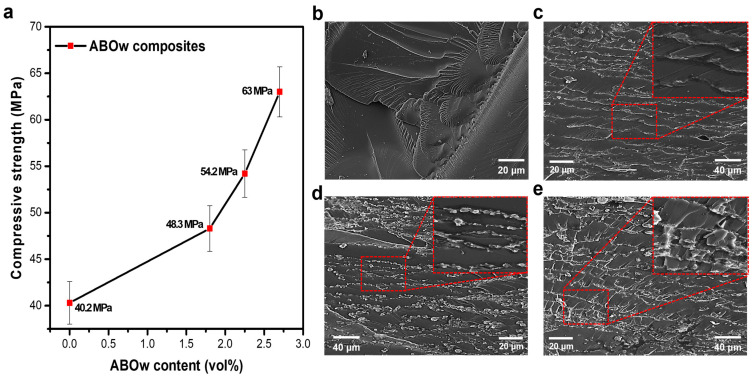
(**a**) Compressive strengths of the aligned ABOw/epoxy composites with different filler contents; (**b**–**e**) FE-SEM images of the fracture surfaces of pure epoxy and composites with different filler contents.

Here, $\sigma_{c}$, F, and A represent the compressive strength (Pa), compressive load (N), and area over which the load acts ($m^{2}$), respectively. Figure 5a shows the compressive strengths of the 3D ABOw/epoxy composites with various filler contents. As the filler content increased, the compressive strength of the 3D composite increased, and the highest compressive strength of 63 MPa was achieved when 2.7 vol% ABOw was added. Although this value is low compared to the absolute mechanical strength of Al and glass, the strength-to-density ratio of the developed composites shows a similar level due to their low density. To analyze the mechanical strengthening behavior of the 3D composites, their fracture surfaces were examined using field-emission scanning electron microscopy (FE-SEM). The pure epoxy without filler easily failed at 40.2 MPa under unidirectional stress (Figure 5b). In contrast, for the 3D ABOw fillers formed in aligned and repeating layers with increasing filler content, the bond strength increased because of their networking of the fillers. This implies that the stress applied to the composite was effectively transferred to the ABOw structure, suppressing cracking. Additionally, because the filler was uniformly distributed within the composite, the stress was also uniformly distributed, increasing the fracture resistance and the compressive strength (Figure 5c–e). Finally, the wall-to-wall distance of the ABOw structure decreased with an increase in the filler content. This indicated that a denser and more constant wall-to-wall distance corresponded to a more significant increase in the density of the added filler at the same volume ratio, resulting in higher fracture resistance; thus, the compressive strength increased significantly as the filler content increased. The composite material with the shortest wall-to-wall distance exhibited the highest compressive strength (63 MPa). In addition, the electrical resistance of the manufactured composite material was measured using a DC source, and it was confirmed that it had an electrical resistance of ≥$10^{12}$ Ω∙cm at 50 °C (Figure S6a). The developed 3D-aligned ABOw/epoxy composite material may be utilized as a protective material for portable electronic devices due to its high mechanical strength, lightweight nature, and excellent insulating properties of epoxy.

## 4. Conclusions

In this study, we developed a 3D-aligned ABOw-based polymer composite material with enhanced mechanical properties for protecting portable electronic devices. We propose a simple method for fabricating uniformly aligned 3D ABOw structures by applying a dispersion method through pH control based on the NaOH content. The resulting 3D ABOw/epoxy composites, which exhibited repeatedly layered and uniformly distributed structures, were obtained after infiltrating epoxy into the 3D-aligned ABOw structure. A composite with a low filler content of 2.7 vol% exhibited a high compressive strength of 63 MPa, representing a 56.72% improvement over pure epoxy. The proposed approach can efficiently improve the mechanical properties of composites with low filler contents. In the future, it would be beneficial to explore not only further optimization of the dispersion method but also the impact of different filler contents and composite geometries on the mechanical performance of this system. Additionally, the thermal, electrical, and environmental resistance properties of the composite may be examined to broaden its applications in other advanced fields. Thus, this composite is expected to be widely used not only for the protection of portable electronic devices but also in the aerospace and automobile industries.

## Data Availability

The original contributions presented in the study are included in the article/Supplementary Material, further inquiries can be directed to the corresponding authors.

## Supplementary Materials

The following supporting information can be downloaded at: https://www.mdpi.com/article/10.3390/ma17194727/s1, Figure S1: (**a**–**d**) turbidity analysis of ABOw slurry with various concentrations of NaOH added (holding time: 0, 5, 10, 15, 20 min); (**e**) Turbiscan stability index (TSI) profiles of ABOw slurries over time at various added NaOH concentration levels; Figure S2: (**a**) Structures composed of slurry content of 0.9 vol%, 1.35 vol% solids, respectively; (**b**) SEM image and EDS mapping of structure fabricated at a slurry content of 2.7 vol% solids; Figure S3: (**a**–**c**) Enlarged SEM images of the structure after heat treatment (×500, ×4000, ×10,000); Figure S4: (**a**) Wall distance graph of 3D structures manufactured with each ABOw content; Figure S5: SEM images of the structures after each heat treatment (500 °C, 600 °C, 700 °C, 800 °C); Figure S6: Graphs of the (**a**) volume resistivity of the ABOw/epoxy composite with various filler contents at 50 °C.
