# Supplementary material for: Enhanced Epoxy Composites Reinforced by 3D-Aligned Aluminum Borate Nanowhiskers
Source: Materials (Basel). 2024 Sep 26;17(19):4727. doi: 10.3390/ma17194727 (PMC11477867; doi:10.3390/ma17194727)
Supplement: Supplementary file 1 [file materials-17-04727-s001.zip › materials-3208813-supplementary.pdf]

Supplementary information

Enhanced Epoxy Composites Reinforced by 3D Aligned Aluminum Borate Nanowhiskers

Hyunseung Song [1,2], Kiho Song [1], Hae jin Hwang [2,*], Changui Ahn [1,*]

[1] Engineering Ceramic Center, Korea Institute of Ceramic Engineering & Technology (KICET), Icheon 17303, Republic of Korea.

[2] Department of Materials Science and Engineering, Inha University, Incheon, 22212, Republic of Korea.

*Corresponding authors: hjhwang@inha.ac.kr (H.J.H.); acu2001@kicet.re.kr (C.A.)

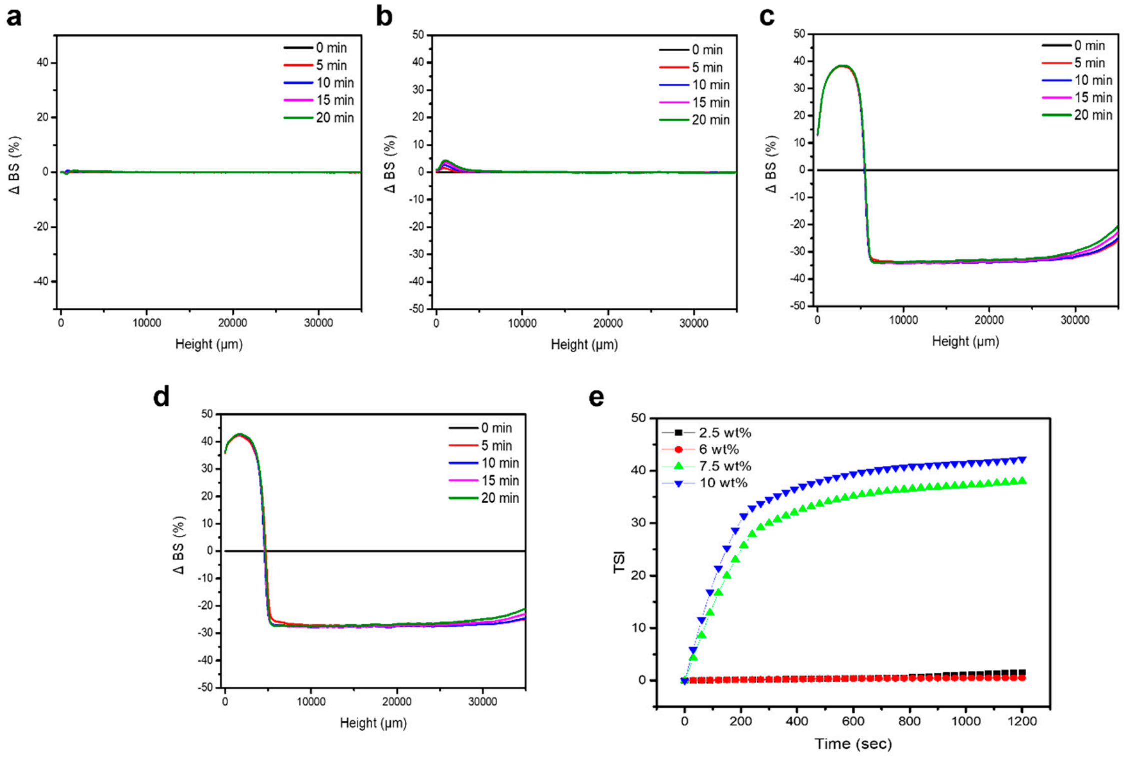

Figure S1 (a-d) turbidity analysis of ABOw slurry with various concentrations of NaOH added (holding time: 0,5,10,15,20 min); (e) Turbiscan stability index (TSI) profiles of ABOw slurries over time at various added NaOH concent levels;

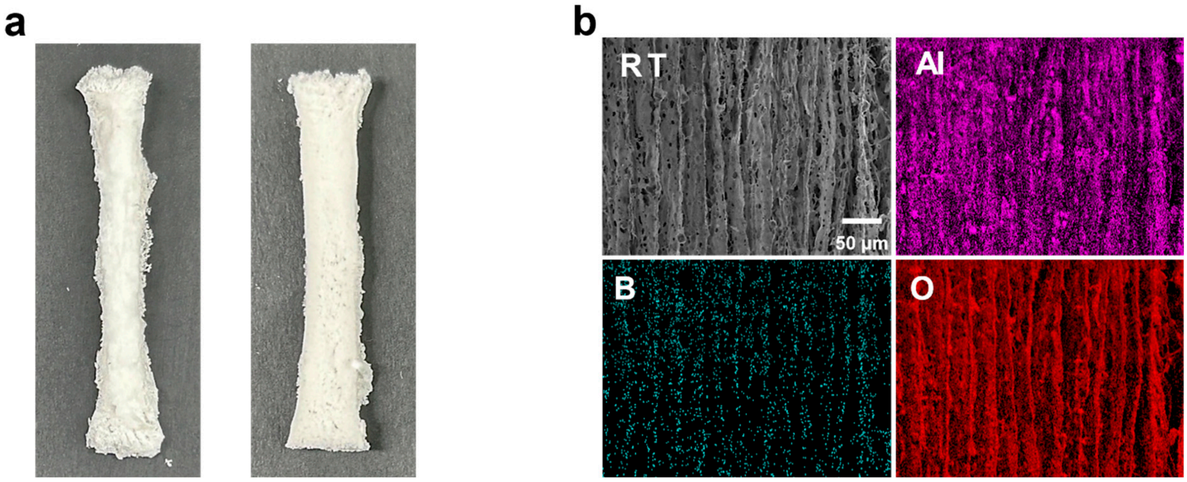

Figure S2 (a) Structures composed of slurry content of 0.9 vol%, 1.35 vol% solids, respectively; (b) SEM image and EDS mapping of structure fabricated at a slurry content of 2.7 vol% solids;

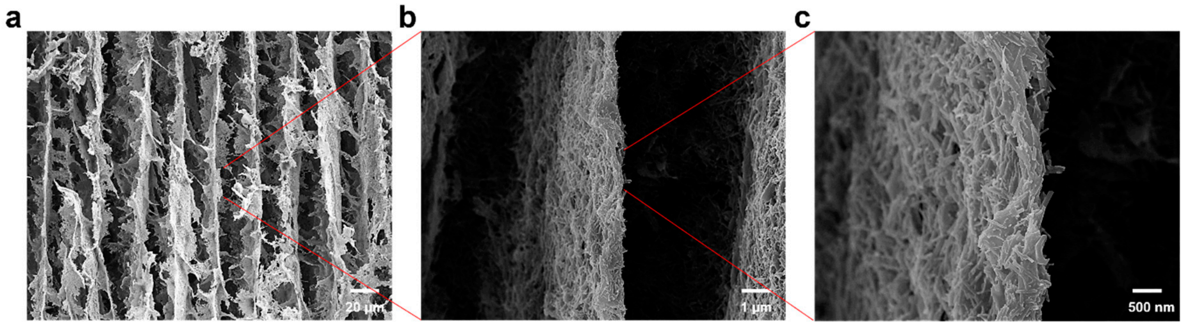

Figure S3 (a-c) Enlarged SEM images of the structure after heat treatment (x500, x4000, x10000);

a

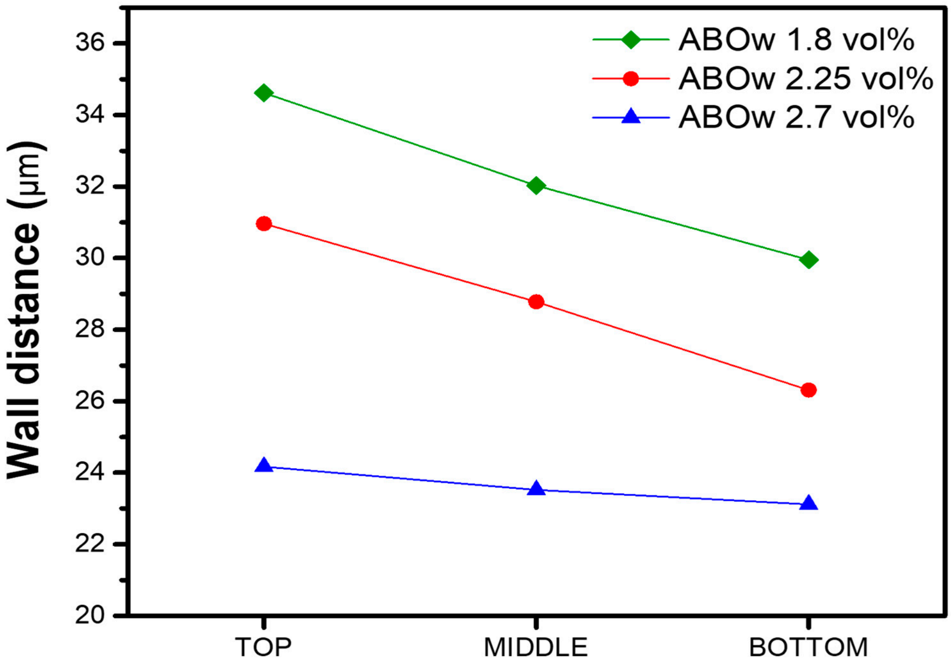

Figure S4 (a) Wall distance graph of 3D structures manufactured with each ABOw content;

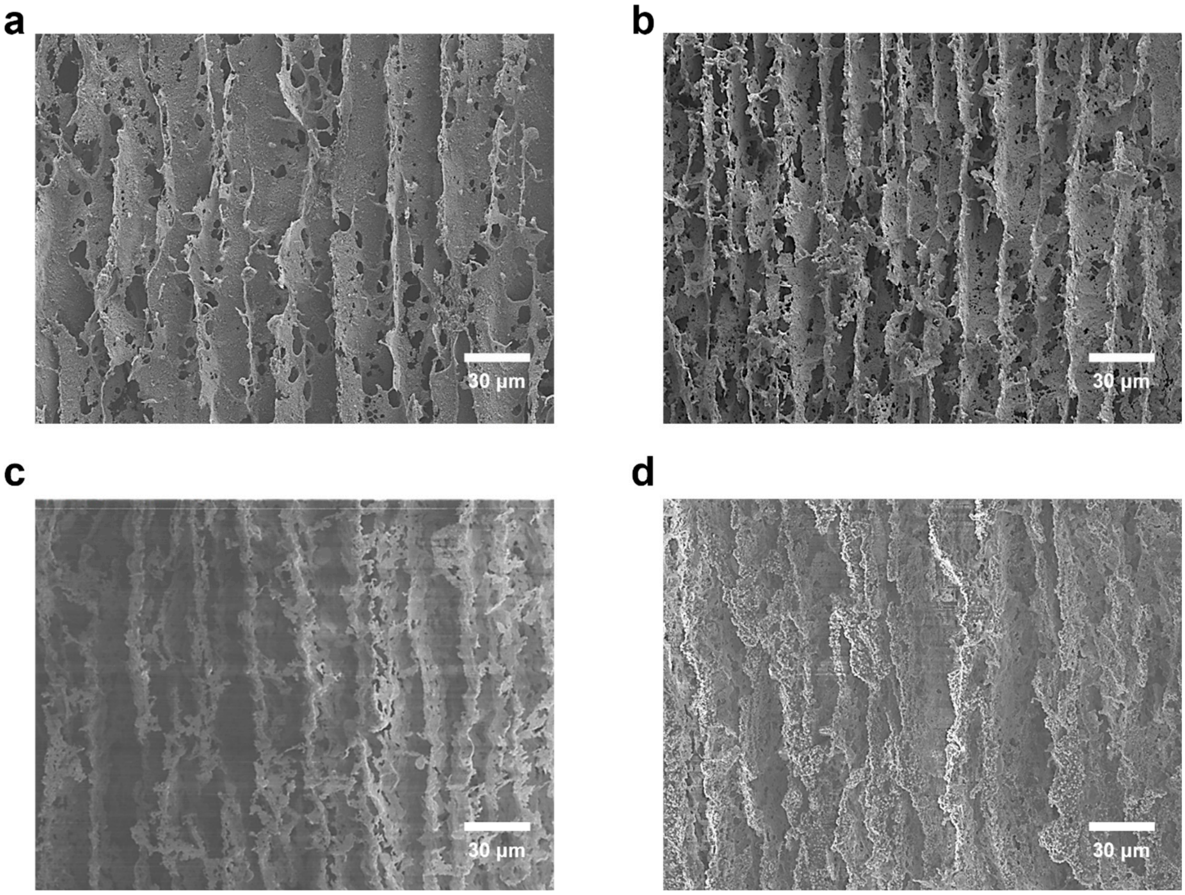

Figure S5 SEM images of the structures after each heat treatment (500 ℃, 600 ℃, 700 ℃, 800 ℃);

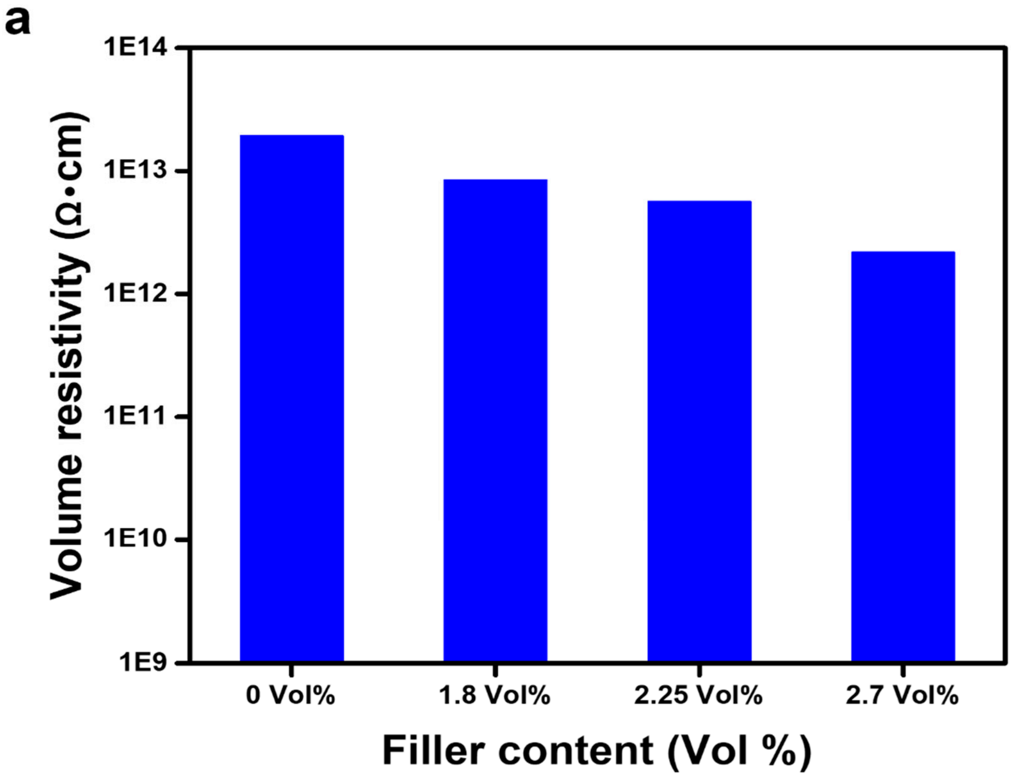

Figure S6 Graphs of the (a) volume resistivity of the ABOw/Epoxy composite with various filler contents at 50 °C;
